# CODE STROKE ALERT—Concept and Development of a Novel Open-Source Platform to Streamline Acute Stroke Management

**DOI:** 10.3389/fneur.2019.00725

**Published:** 2019-07-31

**Authors:** Huey Ming Seah, Moe Burney, Michael Phan, Daniel Shell, Jamin Wu, Kevin Zhou, Owen Brooks, Bronwyn Coulton, Julian Maingard, Jennifer Tang, Gohar Yazdabadi, Bahman Tahayori, Christen Barras, Hong Kuan Kok, Ronil Chandra, Vincent Thijs, Duncan Mark Brooks, Hamed Asadi

**Affiliations:** ^1^Neurointerventional Service – Department of Radiology, Monash Health, Clayton, VIC, Australia; ^2^Deloitte, Sydney, NSW, Australia; ^3^School of Medicine, Monash University, Clayton, VIC, Australia; ^4^Department of Neurology, Monash Health, Clayton, VIC, Australia; ^5^Neurointerventional Service – Department of Radiology, Austin Health, Melbourne, VIC, Australia; ^6^School of Medicine, Deakin University, Waurn Ponds, VIC, Australia; ^7^Alcohol Beverages Australia, Sydney, NSW, Australia; ^8^Department of Engineering, Monash University, Clayton, VIC, Australia; ^9^South Australian Health and Medical Research Institute, Adelaide, SA, Australia; ^10^Department of Radiology, Northern Health, Epping, VIC, Australia; ^11^Department of Neurology, Austin Health, Heidelberg, VIC, Australia; ^12^School of Medicine, The University of Melbourne, Parkville, VIC, Australia; ^13^Stroke Division, The Florey Institute of Neuroscience and Mental Health, University of Melbourne, Parkville, VIC, Australia

**Keywords:** endovascular clot retrieval, stroke, open-source, communication platform, geotagging, mechanical thrombectomy

## Abstract

**Introduction:** Effective, time-critical intervention in acute stroke is crucial to mitigate mortality rate and morbidity, but delivery of reperfusion treatments is often hampered by pre-, in-, or inter-hospital system level delays. Disjointed, repetitive, and inefficient communication is a consistent contributor to avoidable treatment delay. In the era of rapid reperfusion therapy for ischemic stroke, there is a need for a communication system to synchronize the flow of clinical information across the entire stroke journey.

**Material/Methods:** A multi-disciplinary development team designed an electronic communications platform, integrated between web browsers and a mobile application, to link all relevant members of the stroke treatment pathway. The platform uses tiered notifications, geotagging, incorporates multiple clinical score calculators, and is compliant with security regulations. The system safely saves relevant information for audit and research.

**Results:** Code Stroke Alert is a platform that can be accessed by emergency medical services (EMS) and hospital staff, coordinating the flow of information during acute stroke care, reducing duplication, and error in clinical information handover. Electronic data logs provide an auditable trail of relevant quality improvement metrics, facilitating quality improvement, and research.

**Discussion:** Code Stroke Alert will be freely available to health networks globally. The open-source nature of the software offers valuable potential for future development of plug-ins and add-ons, based on individual institutional needs. Prospective, multi-site implementation, and measurement of clinical impact are underway.

## Introduction

Gold standard treatment of acute ischemic stroke relies on timely reperfusion of ischemic cerebral tissue. The two primary methods of reperfusion are thrombolysis using intravenous tissue plasminogen activator (IV tPA) and mechanical thrombectomy. These therapies have been proven to be effective in several randomized clinical trials and meta-analyses (1–3). With rapid reperfusion therapy, up to 70% of patients may have good neurological recovery (4).

Mechanical thrombectomy is superior to IV tPA for acute ischaemic stroke due to large vessel occlusion (LVO). In multiple recent trials, the effect of time to reperfusion with mechanical thrombectomy was directly proportional to patient outcome (5–7). It is estimated that every 1-min reduction in the interval between stroke onset and start of mechanical thrombectomy results in an additional week of healthy living (8).

A comprehensive stroke service is necessarily complex, involving medical professionals across multiple disciplines and departments. Such an intricate system is prone to inefficiencies related to delayed dissemination of information, communication duplication, and error as well as the need for consensus decision making among stroke specialists and neuro-interventionalists (NI). The time critical nature of cerebral reperfusion is a compelling motivation to optimize communication systems, allowing for synchronization and improved coordination of clinical information (9).

To address this need, we aimed to develop a novel, open-source, multi-platform communication application, named “Code Stroke Alert,” that facilitates efficient multi-cast, tiered communication during the hyperacute management stages of an ischaemic stroke.

### Stroke Pathway and Barriers to Timely Reperfusion

**Figure 1:**
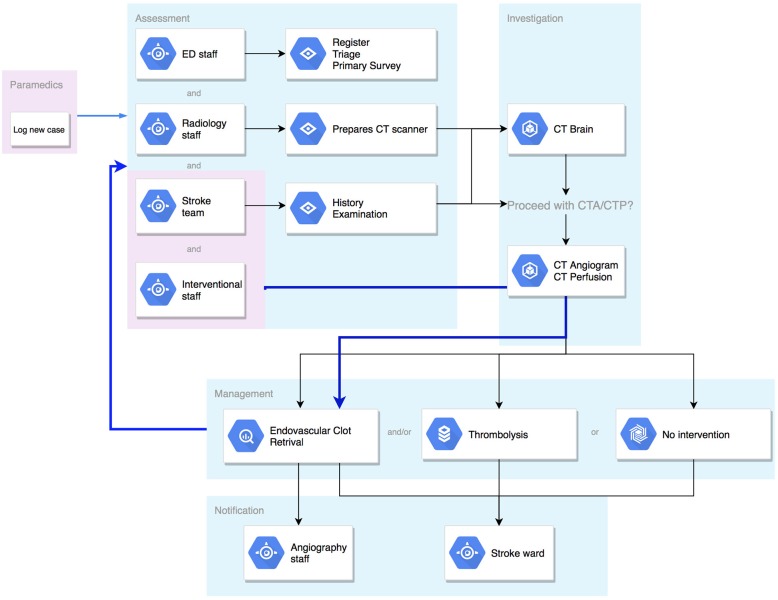
Flowchart of communication pathway in a stroke case.

The basic pathway of communication in a stroke patient is outlined in Figure 1. Most strokes occur in the community and rely on initial recognition by the patient or a family member with subsequent first medical contact with local emergency medical services (EMS). Pre-hospital triage and care varies based on locality but generally involves a basic assessment by trained paramedics and transfer to a local stroke centre. The emergency department (ED) then alerts the radiology and stroke staff of the impending stroke (in most hospitals this is achieved through a group page). Much of the communication to this point is one-way, with limited ability to provide confirmation by any party, nor detailed clinical information.

On arrival to hospital, multiple processes happen quickly and simultaneously. The stroke patient will be reassessed in detail by ED nurses, physicians, and the stroke team, whilst simultaneous handover is provided by the paramedics. Throughout this period, there is constant communication between all parties and senior clinical decision makers within the team. Following clinical assessment, the patient will proceed to neuroimaging with computed tomography (CT). Based on imaging findings in correlation with clinical assessment, a treatment decision will then be made. If the decision is for mechanical thrombectomy, activation of the angiography lab and anesthetics team must occur immediately, with broader notification (stroke unit and admission logistics) shortly after commencement of the procedure.

There are a number of points in this complex process which are vulnerable to delay (Table 1). The initial recognition by community members is the first and there are concerted efforts being made to improve health literacy to address this issue. Once EMS have been contacted there is a triage and dispatch process and then time spent in assessment and information gathering. A decision has to be made regarding destination, followed by transport time to the target hospital. Within the hospital environment there is a further complex succession of medical assessment, imaging acquisition, decision-making and treatment, and in some cases, transfer to a mechanical thrombectomy-capable service may be required.

**Table 1:** Potential systemic barriers to timely reperfusion therapy.

**Community**	**EMS**	**Hospital**
**BARRIERS TO TIMELY REPERFUSION**		
Delayed or mistaken identification of stroke	Delay in arrival or inappropriate triage	**Delay in initial assessment or triage**
Delay in alerting EMS	**Delayed assessment and information gathering**	**Delay in imaging acquisition or review**
	**Delayed decision-making**	**Delayed decision making**
	Transport to hospital	**Delay in activation of mechanical thrombectomy team**
		**Delay in transfer from the emergency department to angiography**
		**Delay in transfer from an ED to another mechanical thrombectomy-capable hospital**

*Those in bold may be addressed by an electronic communications application (9)*.

Up to 40 different health care professionals may be involved in this entire process for an individual stroke presentation. This includes, but is not limited to, paramedic staff, ambulance staff, emergency staff, stroke physicians, stroke nurses, radiologists, radiographers, radiology nurses, NI, anesthetics staff, orderlies, junior medical staff, and administrative staff.

Reliable and efficient communication is a necessity in such a scenario, but most hospitals currently rely on paging systems or paper information sheets with multiple handovers and a sequential dissemination of information. This can lead to communication duplication, information loss, security concerns and, ultimately, time delays. The bold text delays presented in Table 1 highlight points at which we hypothesized that a dedicated, electronic information gathering and distribution program could accelerate the pathway of a stroke patient.

## Methods

### Application Development

The Code Stroke Alert development team consists of a software engineer, a stroke neurologist, NI, a policy advisor, diagnostic neuroradiologists, a neurovascular stroke nurse practitioner, junior medical doctors, and medical students. All team members are volunteers who work collaboratively on different aspects of Code Stroke Alert based on their expertise. Code Stroke Alert development formally commenced in September 2017.

### Development of the Electronic Platform

Given the wide range of healthcare professionals for whom the electronic communications platform is intended, the application has been written and developed by a multi-disciplinary team so that the priorities of each specialty are represented in the application and that user experience is logical and practical. The software developers have a background in healthcare or have experience in developing healthcare-related applications, including familiarity with the Health Insurance Portability and Accountability Act (HIPAA) and importance of privacy with protected health information (PHI).

### Roles of Targeted Audience and Users

**Figure 2:**
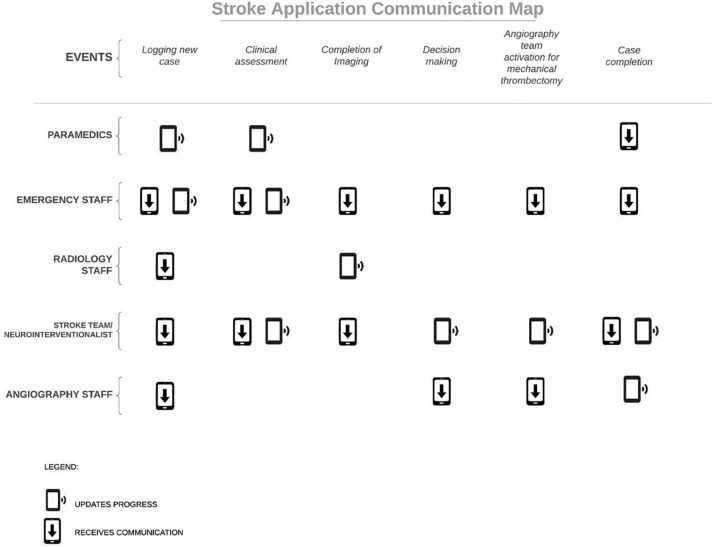
Communication of various events in an acute stroke between different teams with tiered notifications.

The electronic communication platform allows for multiple users to be logged on at the same time, including EMS, ED staff, radiology staff, stroke team, NI, angiography staff, stroke wards, and administrators, given the nature of required simultaneous input of data in the situation of an acute stroke (Figure 2). It provides tiered-level of access to data based on individual staff roles so that patient confidentiality and clinical relevance is maintained throughout the process. It should also allow for revision of incorrect information to maintain accuracy of data that is entered into the application.

The platform is designed with relevant fields of input tailored to individual roles along the care pathway, and each specialty's data logs can be referred to in the future. This facilitates the auditing process, quality assurance, and data collection for research.

### Mobile Platform and Web Browsers

Mobile smartphones are now ubiquitous among health professionals and are crucial for effective communication. Key features of smartphones include the touchscreen, push notifications, and security. Touchscreen interfaces are efficient, intuitive, and highly flexible. Push notifications are alerts which trigger even if the phone is not being used actively. These can be audio or tactile and blend into the background use of the phone. A read receipt system is implemented to close the communication loop between team members. Much more information can also be conveyed than in a traditional paging system. Security features in the iOS (Apple Inc, California, U.S) and Android (Google LLC, California, U.S) systems, two of the most common smartphone platforms, also ensure that the information is stored safely and all applications are routinely reviewed by proprietors to ensure they are free of malicious software.

While mobile devices have many advantages, a secure, computer-based intranet system is also a necessity in modern hospitals. This ensures the protection of an individual health networks PHI through the hospital firewalls and allows for ongoing use and information retrieval even when offline.

### User Interface

**Figure 3:**
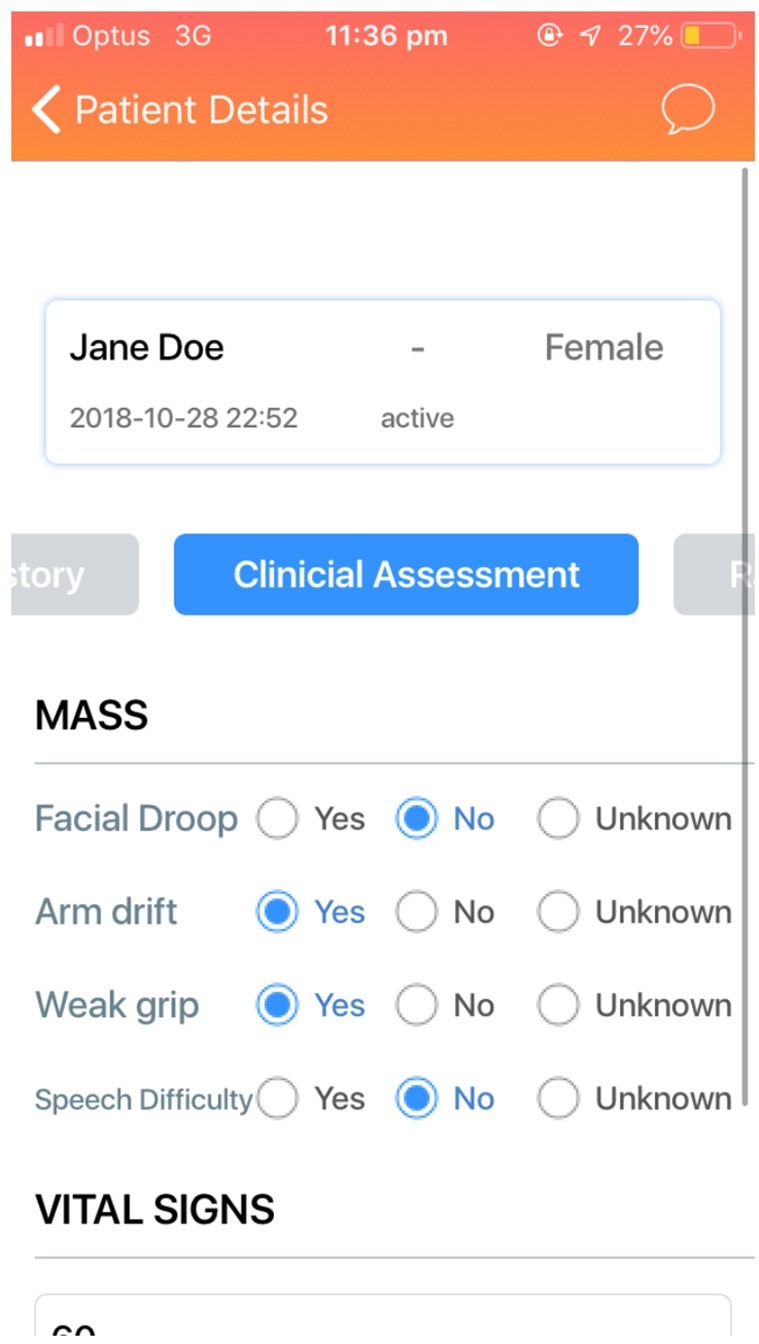
The MASS that has been completed by the paramedics.

An ideal user interface (UI) is intuitive and allows tasks to be completed in an efficient and reliable manner. The Code Stroke Alert platform incorporates clarity, legibility, and comprehensibility to allow for a user-friendly, purpose-built application. This is especially important for a healthcare-related application, anticipating a large number and high turnover of users. The application needs to be intuitive so that new users are able to use the platform effectively without extensive training (Figure 3).

### Tiered Notifications

Tiered notification is a main feature of the electronic communication platform because it allows for users to only be notified with events that are relevant to their clinical specialty and domain of responsibility. The main concern for ongoing and continuous notifications in a busy health network with frequent stroke cases is alarm fatigue. Like instant messaging notifications, if there are too many and frequent notifications, users may stop noticing them.

### Geotagging and Geolocation

Geotagging is a relatively new technology that allows for geographical location of an electronic device to be identified. Code Stroke Alert uses the Google Location application program interface (API) for real-time location tracking while using the application. As such, it is not required for users to run a separate map application for geotagging. This feature is helpful in acute stroke, tracking the patient's location to provide a relatively accurate estimated time of arrival (ETA) with traffic conditions taken into account, to ensure appropriate resource allocation and prioritization, especially in situations where there are multiple impending acute stroke arrivals. Users will first need to allow location detection while using the application when registering for the first time. The physician's location can be geotagged in a way that ETA of the various treating physicians will be available to users, but not the accurate location for privacy purposes. This ensures that other relevant users are informed with the ETA of on-call specialists and can prepare the patient. This function can also be switched off by individual users if desired.

### Security

The most widely used security regulation is the HIPAA, which is a set of rules that dictate the requirements of a healthcare-related application to ensure the three fundamental security goals of confidentiality, integrity, and availability are maintained during transfer of patient information. To help identify possible risks for data breaches or privacy violation, regular audits of the application will be performed. An effective mechanism to communicate errors and bugs in the application between users and developers has been established.

Multi-factor authentication is increasingly used to ensure security of individual accounts. Traditionally, login credentials are made up of a username and a password. However, passwords are becoming easy targets for theft especially when users have weak passwords, the same passwords for multiple applications and have passwords unchanged for long periods of time. Multi-factor authentication in Code Stroke Alert provides an extra layer of security by requesting additional evidence of user credentials.

## Results

We have developed Code Stroke Alert, an HIPAA compliant, open-source platform to streamline and synchronize the flow of clinical information, as it is communicated between different members of health care services during the pre-hospital, in-hospital, and inter-hospital care of patients with acute ischaemic stroke.

### Roles of Targeted Audience and Users

Code Stroke Alert is intended to cover the entire journey of a stroke patient from the initial contact with a healthcare provider, until return to the ward following treatment completion. We first highlight the professionals involved and how the application is intended to streamline their role in the management of a stroke patient, based on the Australian Health care context.

#### Paramedics

Paramedics are responsible for identifying and logging most new, suspected stroke cases. Once they have assessed the patient, they are able to log in to Code Stroke Alert and create a new case by inserting basic demographic information, symptoms, and time of onset. There will be other optional fields available including vital signs, weight, or the Melbourne Ambulance Stroke Screen (MASS), next of kin details and a photo of the patient's identification card. This information is broadcast simultaneously to all relevant team members. The paramedics subsequently receive confirmation of receipt, closing the loop of communication. All of the information provided will help facilitate timely assessment and processing upon arrival at the emergency department.

#### Emergency Department Staff

The ED staff consists of administrative staff, nurses, including those in triage and the main department, and physicians. They are responsible for priority registration of the incoming stroke code, rapid triage, allocation of an appropriate bed, adequate intravenous cannulation, initial blood tests, and facilitation of rapid transfer to the CT scanner. In the event that the patient presents directly to ED rather than via EMS, the ED staff replace the role of the paramedics. The registration details are completed in Code Stroke Alert to ensure nothing is overlooked and completion of these events is updated in the patient file in real-time for all team members, ensuring provision of contemporaneous information and progress.

#### Radiographers

When the radiographers receive notice of the stroke code they can pre-register the patient with the information provided, allowing them to proceed directly to scanning upon arrival. Once the relevant imaging studies are completed, the radiographers can notify the radiologist and stroke team with a simple check button, as well as inform them of any obvious abnormalities. When imaging is completed, another notification can be sent to all relevant parties to facilitate transfer, either to the angiography lab or back to the ED.

#### Diagnostic Radiologist

The diagnostic radiologist receives early alert of the estimated time and actual arrival of the patient in ED. This allows them to prioritize review of the images and assess for intracranial LVO, intracranial hemorrhage (ICH), or perfusion abnormality. These findings can be broadcast via Code Stroke Alert to the rest of the team and in particular, to the NI if LVO is suspected.

#### Stroke Team

The stroke team variably consists of a consultant neurologist, nurse practitioner, and/or junior medical doctors. The stroke team receives early notification of an arriving stroke via Code Stroke Alert. Occasionally, the stroke team will need to log a new case, for acute inpatient stroke events. The application also allows for review of the clinical information gathered by paramedics or emergency staff which can be especially helpful in triaging multiple, simultaneous cases. The stroke team can also easily disseminate the National Institutes of Health Stroke Scale (NIHSS) score and other relevant history or examination findings via Code Stroke Alert. Based on the imaging findings and discussion with the diagnostic radiologist, the stroke team will make a decision on the next step in management which may include mechanical thrombectomy, IV tPA or no intervention. The relevant teams should have been forewarned via Code Stroke Alert, enabling rapid deliberation and transfer from the CT scanner. Finally, the stroke team is informed of the completion and outcome of mechanical thrombectomy and the patient's destination on the ward where they can be reviewed post-procedure.

#### Neuro-Interventional Team

The neuro-interventional team consists of NI, radiology (angiography) nurses, and radiographers.

**Figure 4:**
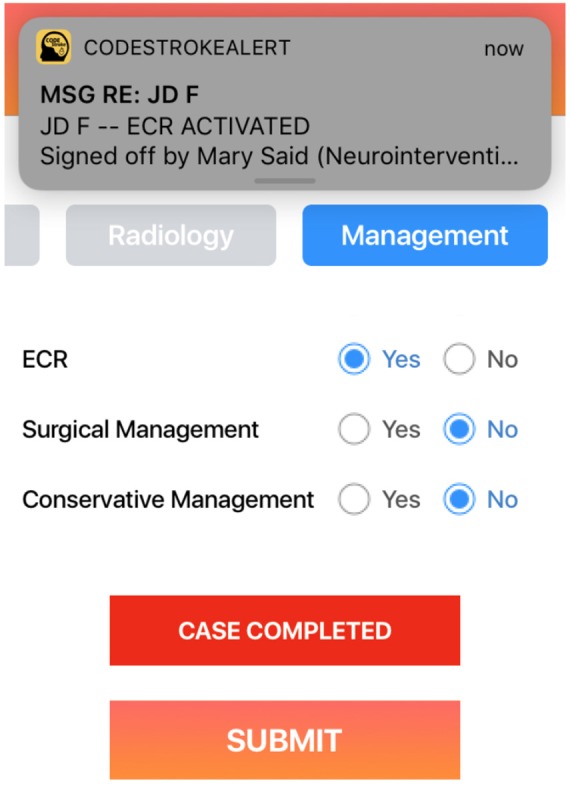
The NI who has been constantly updated with the patient's journey can review the images and activate the mechanical thrombectomy team.

NI will be able to follow the progress of a stroke case via Code Stroke Alert, allowing for early independent or collaborative review of the imaging. If mechanical thrombectomy is viable they will be notified early by alerts in the application and can activate the angiography staff (Figure 4). Upon completion of the case, relevant information regarding imaging outcomes including reperfusion grading, complications, and post-procedure instructions can be transmitted via Code Stroke Alert.

Angiography staff will be pre-notified of a new stroke case and can follow the progress in the application. If the case proceeds to mechanical thrombectomy, the team can be activated via a simple command in Code Stroke Alert. Clinical information can also be reviewed prior to commencing the case and the record kept in the application facilitates handover between nurses and anesthetics team. If the patient is not to proceed to angiography, the staff will also receive a notification to that effect.

#### Anesthetics Team

The anesthetics team consists of the anesthetist and anesthetic nurse, who attend stroke cases during mechanical thrombectomy for airway and anesthetics support. For each case, a decision is made whether intubation is required for the stroke patient and choice of sedation to administer.

Allocation of the anesthetics team is coordinated by the anesthetist-in-charge and the theater floor coordinator. Involvement throughout the communication process aids with triaging and pre-planning of anesthetic resources.

#### Stroke Ward

The stroke ward receives early notification when a new case is logged and when the management for the patient has been decided. The acuity of the patient and treatment pathway will have strong bearing on the ultimate destination. Typically, if the patient has a stable airway after extubation, care on a specialty stroke unit will suffice. A patient who cannot be extubated at the completion of mechanical thrombectomy treatment requires admission to ICU. The ease of communication afforded by Code Stroke Alert facilitates these bed management decisions and overall hospital patient flow.

#### Administrators

The administrative team consists of the Information Technology (IT) staff at individual hospitals to provide support for users of Code Stroke Alert. They are able to access bug reports and customize the application to meet the unique requirements of each hospital. They can also modify plug-ins and add-ons with Picture Archiving and Communication system (PACS) or hospital information system (HIS) integration. Finally, administrators are able to access the database and export data for research and audit purposes.

#### Mobile Platform and Web Browser

Code Stroke Alert is designed for integrated use between a web browser and mobile application. There are a number of differences between the two systems, primarily in remote access, forced upgrades and bug reports. Firstly, users can only access Code Stroke Alert on a web browser on a computer within the hospital network. Remote access is only allowed on mobile phones. This is necessitated as a matter of security for the web browser which does not have the same in-built security of a phone. The second difference between using Code Stroke Alert on a web browser compared to mobile phone application is in regards to software updates. As it is more common for automatic mobile phone upgrades to be turned off, Code Stroke Alert forces users to upgrade to the latest version of the application on their mobile phones whenever a new version is available. Forced upgrading of Code Stroke Alert with minimal frequency is important to reflect bug fixes and security patches, so that the application is safer for users. Upgrading is not required for Code Stroke Alert on web browsers as the application is automatically updated when the website is refreshed. Finally, when an error occurs with Code Stroke Alert, mobile application users are able to report errors by shaking the device which will automatically forward an error report to the software developers. Feedback on web browsers needs to be manually reported via email to the development team.

#### Programming Language

Code Stroke Alert uses the programming language Python (Python Software Foundation, Delaware, U.S.) for back-end programming. The back-end is important for data processing and allows information to be requested and sent through to the users. The server is then updated with the latest data that is received through input by users on Code Stroke Alert, allowing all users to have access to updated information about the patient.

Python is a very popular programming language within the medical and scientific community and was selected for its up-to-date and well-maintained libraries and flexible modules, frameworks and packages. This allows for a greater degree of customization to meet the needs of individual health networks.

The database where Code Stroke Alert stores information is written in the programming language MySQL (Oracle Corporation, California, U.S.). MySQL is an open-source Relational Database Management System (RDBMS) that uses Structured Query Language (SQL). SQL is used for adding, accessing, and managing content in a database, and is noted for its quick processing, flexibility, reliability, and ease of use.

The front-end is the part of the application that users interact with directly. The front-end of the iOS version of Code Stroke Alert is written in the programming language Swift (Apple Inc., California, U.S.), which is a relatively new open-source programming language developed and supported by Apple. The front-end of the Android version of Code Stroke Alert is written in the programming language Java (Oracle Corporation, California, U.S.), similar to the language that was used to write Android itself. It is a very popular and reliable language for writing Android applications and has been established for more than 20 years.

#### User Interface

After initial discussion regarding the functionality requirements and information architecture, the initial wireframes were developed on *Balsamiq* (Balsamiq studios, California, U.S). Feedback from health professionals was then sought to review button choices, sizes, information flow, wording, input controls, and other elements of the user interface.

Free text entry boxes are selected for data fields with unlimited possible input such as patient name, address, phone number, clinical history, past medical history, and medication lists so that data can be entered freely on the platform. Data with limited possible input such as gender are listed as options for easy selection. Similarly, the options for each component of clinical scores are also listed as options so that calculation of the score is possible. Fields with numerical data such as time last seen well, date of birth, blood pressure, heart rate, weight, and oxygen saturation are entered as a scrollable list of numbers.

To expedite data entry, commonly prescribed anticoagulants are listed together with the medication text box that can be ticked if the patient is currently on a particular anticoagulant given its clinical relevance. Relevant past medical history including hypertension, diabetes, previous strokes and ischaemic heart disease are also listed together with the past medical history free-text box.

The final wireframes were developed after an iterative back-and-forth process. A straightforward design and a minimal and concise approach were adopted for the user interface of Code Stroke Alert to optimize usability of the application.

#### Content

Even with an efficient touchscreen input, reliable backend programming and an intuitive UI, every extra piece of information which requires input into Code Stroke Alert is time spent away from assessing and managing the patient. Content selection has been balanced to ensure adequate vital information is entered, whilst minimizing downtime and user fatigue.

For emergency services, some essential information is required, including patient identification, height and weight, destination hospital, and basic presentation including vital signs and last time the patient was seen well. Some aspects of the patient ID can be automatically derived from the mobile phone camera and an ID card and destination hospital automatically defaults to the geographically closest hospital. The remainder of the EMS interface involves clinical history and assessment. Here, relevant and common medical issues such as ischaemic heart disease, diabetes, previous strokes and any anticoagulant use can be easily selected using yes/no checkboxes, and there is additional, non-mandatory, free text input area for other information the paramedics deem to be important.

The MASS, a screening tool currently used by Ambulance Victoria, is included in Code Stroke Alert to aid with diagnosis of stroke. While it is useful, it is not a mandatory input, as this assessment can be complex, and the result will not alter the need for imaging. A prompt is also included to insert an 18G intravenous cannula to facilitate contrast power injection for CT angiographic stroke imaging upon arrival at hospital, or subsequent thrombolysis. This is an important step which can delay imaging, thus justifying its specific inclusion as a prompt. A summary page is also included, providing a quick review of the information provided, which can be performed in transit.

For hospital staff Code Stroke Alert is divided into a page detailing all incoming, active, and completed stroke codes which lead to the individual patient pages containing their details. Often multiple stroke codes occur concurrently and several can occur in a day. It was deemed necessary to have a quick way of listing all current stroke patients, differentiating between them, and tracking their general progress. The information collected within Code Stroke Alert is derived from that which is deemed necessary by physicians and non-clinical staff to provide management in hospital. Within the individual patient pages, current location and primary survey data are followed by the EMS, and finally sections for in-hospital assessment and management. This last section includes NIHSS, modified Rankin score (mRS) and tabs to facilitate imaging and communicate treatment decisions. The NIHSS allows for baseline comparison pre- and post-treatment. The mRS guides the ceiling of treatment that would be offered to patients. The imaging information is especially important when multiple CT scanners are in use. Completion of the scan and early diagnostic results can be quickly communicated with checkboxes in the individual patient page, as can the decision for thrombolysis, mechanical thrombectomy, or conservative management.

#### Tiered Notifications

The notifications broadcasted by Code Stroke Alert are filtered and tiered to prevent alarm fatigue. The notifications are tailored to individual roles so that users only receive relevant notifications. For example, when the CT is marked as completed by the radiographer, the notification is sent to the stroke team, the NI and the diagnostic radiologist but not to ED staff, the angiography team or the stroke ward as these staff are not required to review the images immediately to devise a management plan. By filtering the notifications and only activating physicians at appropriate timepoints, alarm fatigue can be reduced and user satisfaction can be maintained. Users are also able to unsubscribe to notifications for a particular case if they do not wish to receive alerts about a particular patient but still want to be informed of the outcome of the case. For example, this would be relevant for a Stroke consultant who may not be on-call that day but will be looking after the stroke patients the following day. Ultimately, the system may be adapted to the needs of each health network, team, and individual.

#### Other Features

Code Stroke Alert has been designed to provide assistance to all parties involved in acute stroke care through a number of carefully selected features.

Geolocation and geotagging allows for the automatic recommendation of the nearest and most appropriate hospital and for real-time location to be broadcast to all team members. The estimated time of arrival of both the patient and NI, if they are off-site, can also be automatically broadcast, although the exact location of the NI is withheld for privacy.

As discussed, multiple clinical score calculators such as the NIHSS, MASS and mRS are integrated into Code Stroke Alert. They provide useful predicative information and have been designed within the application to allow for ease of tabulation. Automatic clinical prompts are also designed to popup based on input information such as warnings against thrombolysis if anticoagulation is noted. While they are useful, the risk of alarm fatigue and excessive prompting is recognized and Code Stroke Alert is designed to be modular to allow for individual health networks and teams to tailor the system to their preference.

In addition, Code Stroke Alert provides a secure communication platform between all users. The platform allows for instant messaging with read receipts to prevent breakdown of communication and avoid duplication. It also allows for photos and videos to be shared within the chat group if required. This feature can be particularly helpful for recording the patient's neurological deficit or recording the acquired CT images if some physicians are off-site and do not have direct access to Radiology or are not able to examine the patient themselves.

In the event that the users of Code Stroke Alert do not have their mobile phones with them, there is an option to integrate its notifications with the conventional paging system. In such cases, the tiered notification system will be applied to the paging system.

Code Stroke Alert is also open source making the application easily customizable to adapt to the individual needs of the health network or team. Synchronization with PACS and HIS systems is also possible providing automatic access to radiology and the patient's medical record via Code Stroke Alert.

Furthermore, all data entered into Code Stroke Alert are timestamped and securely stored. The collected data is useful for quality improvement and research purposes. The users of Code Stroke Alert can share this information easily and securely with other hospitals if they choose to provide efficient handover to physicians from other health networks, and to assist in collaboration and improving overall patient care.

Finally, to assist with the debugging process in the trial phase of Code Stroke Alert, users are able to log a bug report by just shaking their mobile phones which will create a free text pop-up box which will feed back directly to the development team. The bug report captures the actual moment and page that users are experiencing problems. It is designed to be as simple as possible to maximize the chance busy healthcare professionals will report issues.

### Security and Legal Compliance

Code Stroke Alert is fully compliant with the HIPAA. The platform uses secure sockets layer (SSL)/transport layer security (TLS) encryption and decryption for all data transfer requests, which is a security protocol to ensure data encryption, integrity and authentication. This is to prevent sensitive PHI from being leaked to unauthorized parties.

All users will have a login page for security reasons. To strike a balance between security and user fatigue, a single login is required every 24 h. Code Stroke Alert uses multi-factor authentication to increase the security and protect users of the platform.

Code Stroke Alert has a decentralized database and has pieces of information stored in different locations which are all connected to each other. With a decentralized network and data distributed amongst multiple nodes, all data is backed up on multiple nodes and constantly protected if any data is maliciously altered. This eliminates vulnerability to hacking as all of the nodes that hold the data would have to be attacked at the same time to destroy data on the decentralized network. It also prevents accidental change of data. Furthermore, in the event of compromise of any individual node, the remaining nodes can retain function with data transfers as the damaged node is being replaced or repaired.

Lastly, Code Stroke Alert is made secure by storing the database within the server of the health network. It is kept as secure as possible by the existing firewall and demilitarized zone of the hospital's network.

## Discussion

Code Stroke Alert is a digital platform designed to streamline the communication process in the acute management of stroke. It is a user-friendly, purpose-built application that offers convenient means of data entry and sharing.

There is an increasing use of mobile devices and applications by health care providers to assist with many important tasks, including information and time management, health record maintenance and access, communications, information gathering, patient management and monitoring, clinical decision making, and medical education and training (10). In a survey of healthcare providers and medical students, more than 80% of respondents described using e-mail, telephone, or text messages to communicate with colleagues about patient care, as it is described to be a more efficient way of communication than meeting in-person or having a phone conversation (11).

Usage of mobile applications have provided many benefits to health care providers, such as convenience, increased efficiency, and enhanced productivity. Code Stroke Alert is convenient for users as it is able to be accessed through hospital computers or personal devices. It allows users to utilize the application while they are not on hospital grounds. Code Stroke Alert also increases efficiency by allowing clinicians to triage and plan their time around the arrival of these stroke cases and to allocate resources effectively based on clinical urgency.

The use of mobile applications during delivery of acute stroke care is well-known in stroke literature. The Join application (Allm, Inc, Tokyo, Japan) and Stop Stroke (Pulsara, Montana, U.S.) are two of the several existing communication applications to enhance the coordination and communication in delivery of acute stroke management (12, 13). Table 2 outlines the features that are offered by Code Stroke Alert, Join application, and Stop Stroke.

**Table 2:** Features of Code Stroke Alert, Join application, and Stop Stroke.

**Features**	**Code Stroke Alert**	**Join application**	**Stop Stroke**
Pre-hospital notifications	✓	✓	✓
Comprehensive intra-hospital notifications	✓	✓	✓
Inter-hospital notifications	✓	✓	✓
Open source	✓	✗	✗
Customisable	✓	✗	✗
Data collection	✓	✓	✓
Geolocation	✓	✓	✓
Secure messaging	✓	✓	✓
Full iOS, android and web browser functionality	✓	✗	✗

To our knowledge, Code Stroke Alert is the first stroke communication application that allows individual health networks to customize the application to their needs through an open source feature.

It is also the first stroke communication application that can be used on web browsers as there are staff involved in the stroke communication pathway who are normally stationed at a particular computer throughout their shift. Allowing Code Stroke Alert to be used on web browsers prevents these staff from having to swap between their computers for work purposes and mobile phones to check Code Stroke Alert notifications.

### Current Progress and Utility

Code Stroke Alert is currently approved for trial at two Comprehensive Stroke Centers in Australia. This trial follows approval from multiple services in these centers, including Emergency, Radiology, Stroke, NI, and Information Technology Departments.

The effectiveness of the platform will be evaluated using a pre-post historical design to study the impact of Code Stroke Alert implementation upon time to reperfusion therapy. In these facilities, detailed treatment and outcome records for comparison. Further improvements will be made to the platform based on user feedback prior to trialing Code Stroke Alert on a larger scale using a stepped wedge cluster randomized trials.

The initial trial setting will also be used to actively monitor and remove glitches and bugs and to implement further user-friendly features, with the goal of seamlessly integrating the application with day to day stroke unit work flow, tracking a patient through their entire hospital journey. There is ongoing designer and developer support for integration with the individual hospital PACS and HIS, as well as relevant EMS applications and programs, with the intension of optimizing the usability of the platform.

The final goal of the trial process is to further develop the application into a stand-alone application that is free to download and implement easily in stroke units around the world without the need for developer installation, extensive support, and troubleshooting.

### Research Potential

Data entered into Code Stroke Alert are saved into a secure, de-identified database on the hospital's network. This rich database with archived data from the hospital's stroke cases can be audited and analyzed in the future for quality improvement, for planning appropriate service funding, resource allocation and service development, expansion of facilities and for research purposes.

## Conclusion

Code Stroke Alert is a new, user-friendly, purpose-built application to streamline hyperacute stroke management. It incorporates high-level mobile-oriented features like geolocation and push notifications to improve multidisciplinary communication. After testing is complete, the back-end coding for Code Stroke Alert will be made freely available to researchers globally, with potential for customization to meet the needs of individual health networks and widen the scope to other time-critical diseases.

## Data Availability

All datasets are included in the manuscript.

## Author Contributions

HS, MBu, BC, JM, RC, VT, MBr, BT, and HA contributed conception and design of the application. MBu, MP, DS, JW, OB, and JT developed the application. HS and KZ wrote the first draft of the manuscript. HS, KZ, GY, CB, and HK wrote sections of the manuscript. All authors contributed to the manuscript revision, read, and approved the submitted version.

### Conflict of Interest Statement

MBu is a current employee of Deloitte Australia and GY is a current employee of Alcohol Beverages Australia.

The remaining authors declare that the research was conducted in the absence of any commercial or financial relationships that could be construed as a potential conflict of interest.
